# Effective Treatment of Lung Adenocarcinoma With a Novel SLC44A1-BRAF Fusion Using Pembrolizumab Followed by Trametinib: A Case Report

**DOI:** 10.7759/cureus.54739

**Published:** 2024-02-23

**Authors:** Sho Yasui, Takayuki Honda, Iichiro Onishi, Sadakatsu Ikeda, Yasunari Miyazaki

**Affiliations:** 1 Department of Respiratory Medicine, Tokyo Medical and Dental University, Tokyo, JPN; 2 Department of Pathology, Tokyo Medical and Dental University, Tokyo, JPN; 3 Center for Innovative Cancer Treatment, Tokyo Medical and Dental University, Tokyo, JPN

**Keywords:** slc44a1, trametinib, pembrolizumab, braf-fusion, lung adenocarcinoma

## Abstract

The serine-threonine protein kinase B-RAF (BRAF) fusions are rarely observed in non-small cell lung cancer (NSCLC) accounting for less than 1%, and therapeutic evidence for molecular-targeted drugs is lacking, unlike for BRAF V600E mutation by RAF and MEK inhibitors. A 75-year-old female patient with no smoking history and mild renal dysfunction developed recurrent lung adenocarcinoma and was initially treated with pembrolizumab immunotherapy followed by chemotherapy using docetaxel showing a certain efficacy but the disease finally progressed. Comprehensive genome profiling showed a novel SLC44A1-BRAF fusion and the tumor progression was controlled with the MEK inhibitor trametinib. Because of the rarity of NSCLC with BRAF fusion, the description of this case would be helpful for the treatment strategy for such tumors.

## Introduction

The serine-threonine protein kinase B-RAF (BRAF), encoding an RAF kinase, plays a pivotal role in activating the MAPK pathway. Aberrations in the BRAF gene serve as oncogenic drivers in a variety of solid tumors, notably non-small cell lung cancer (NSCLC), while also presenting as viable therapeutic targets [1]. BRAF mutations are identified in approximately 1-4% of NSCLC cases [1-3], with the V600E point mutation being the most prevalent subtype, accounting for over half of NSCLC cases with BRAF mutations [4]. Patients with NSCLC harboring the BRAF V600E mutation have demonstrated significant clinical improvement following combination therapy with dabrafenib and trametinib [5]. Nevertheless, there is a lack of established targeted therapies for NSCLC patients with non-V600E BRAF mutations [3].

Despite their relative scarcity, accounting for only 0.2% of cases, BRAF fusion gene aberrations in NSCLC have been detected through comprehensive genome profiling (CGP) [6]. The therapeutic approach for tumors with BRAF fusion aberrations contrasts with that for the BRAF V600E mutation. In a phase 2 study, first-generation BRAF inhibitors, such as sorafenib, proved ineffective in treating BRAF fusion malignancies [7], a finding potentially attributable to the distinct oncogenic properties of the BRAF fusion protein compared to the V600E mutation [8]. Only a minority of cases have responded positively to treatments involving trametinib and/or sorafenib [6,9,10]. Consequently, there is an urgent need to expand our understanding and treatment modalities for BRAF fusion tumors.

Here, we report a case of NSCLC with a novel SLC44A1-BRAF fusion, where the patient's tumor responded favorably to an immunotherapeutic regimen involving pembrolizumab, followed by trametinib treatment.

## Case presentation

**Figure 1:**
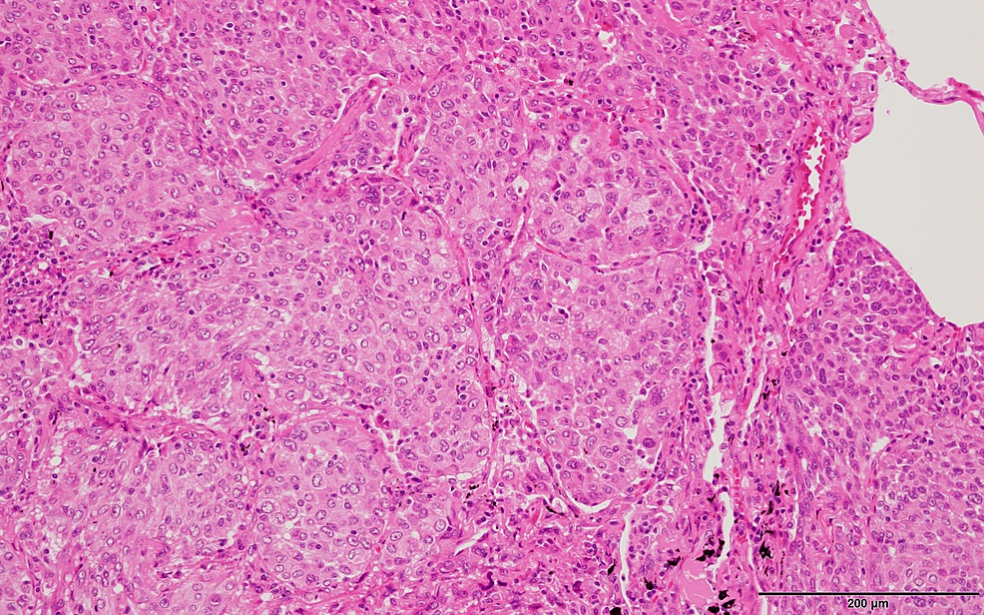
Hematoxylin-eosin stain of surgically resected specimen (100 x).

A 71-year-old female patient with no history of smoking was referred to our hospital for a nodule in the right lower lobe revealed by chest computed tomography (CT). The clinical presentation was suggestive of lung cancer, leading to her undergoing a thoracoscopic lobectomy of the right lower lobe along with lymph node dissection. Pathological examination confirmed the diagnosis of adenocarcinoma (pT2aN2M0, Stage IIIA, TNM 8th edition), characterized by atypical epithelial cells with pale nuclei and acidophilic cytoplasm proliferating in solid nests, coupled with mild lymphocytic infiltration within the tumor (Figure 1).

She received adjuvant chemotherapy, consisting of four cycles of cisplatin (CDDP 80 mg/m2, Day 1) and vinorelbine (VNR 25 mg/m2, Day 1 and 8).

**Figure 2:**
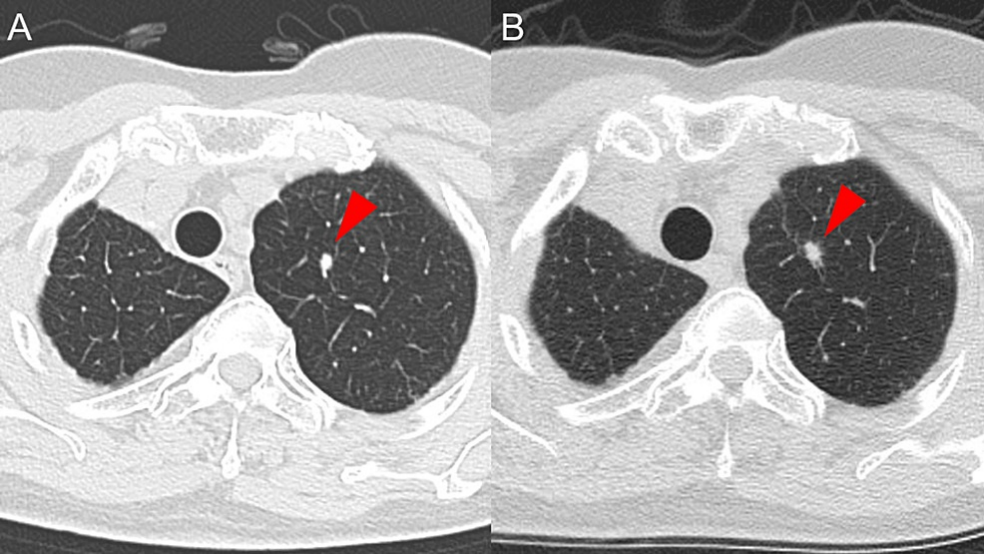
A series of chest computed tomography during pembrolizumab treatment; at the initiation (A) and nine months after the initiation of the treatment (B), showing stable disease.

Four and a half years post-surgery, a follow-up CT scanning revealed metastases in the lungs, lymph nodes, pleura, and bones. Oncomine™ Dx Target Test® did not identify any targetable mutations, but immunostaining indicated a positive programmed death-ligand 1 (PD-L1 clone, 22C3, Agilent) tumor proportion score (TPS 1-24%) using surgical samples. The patient was then started on first-line immunotherapy with pembrolizumab (200 mg/body, every three weeks) because of her elderly age and renal dysfunction. A CT scan conducted nine months after starting pembrolizumab treatment showed stable disease with slight tumor progression (Figure 2A, 2B).

However, after 17 cycles, the pembrolizumab treatment was deemed unsuccessful. Subsequently, second-line chemotherapy with docetaxel (80mg/m2) was administered for a total of 22 cycles with dose adjustments, but the disease eventually became refractory to docetaxel.

**Figure 3:**
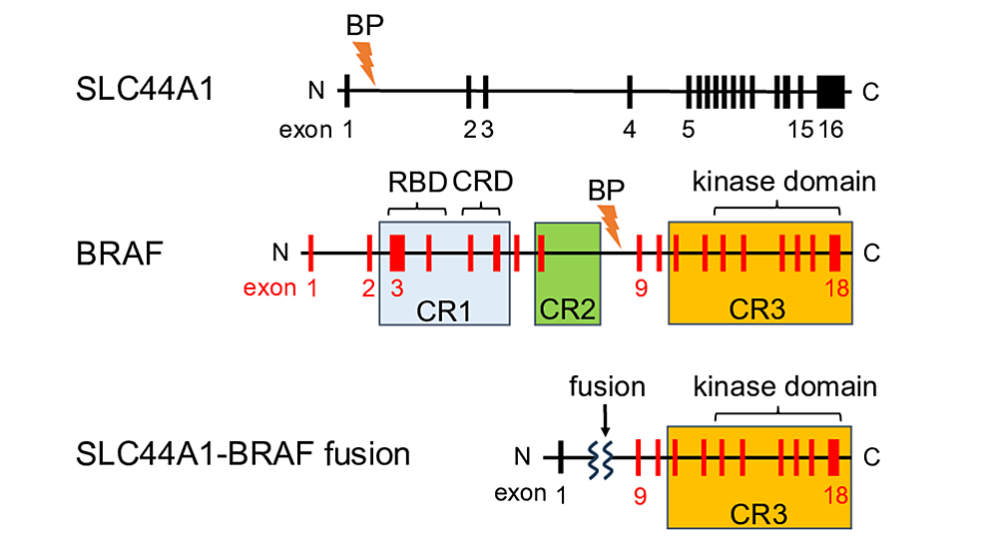
A schematic illustration of SLC44A1, BRAF, and SLC44A1-BRAF fusion detected in this case. BP: breakpoint, CR: conserved region, RBD: RAS-binding domain, CRD: cysteine-rich domain

In search of potential therapeutic targets, a CGP test using FoundationOne® CDx on a formalin-fixed paraffin-embedded (FFPE) sample from the surgery was conducted. This test uncovered a novel SLC44A1-BRAF gene fusion (Figure 3).

**Figure 4:**
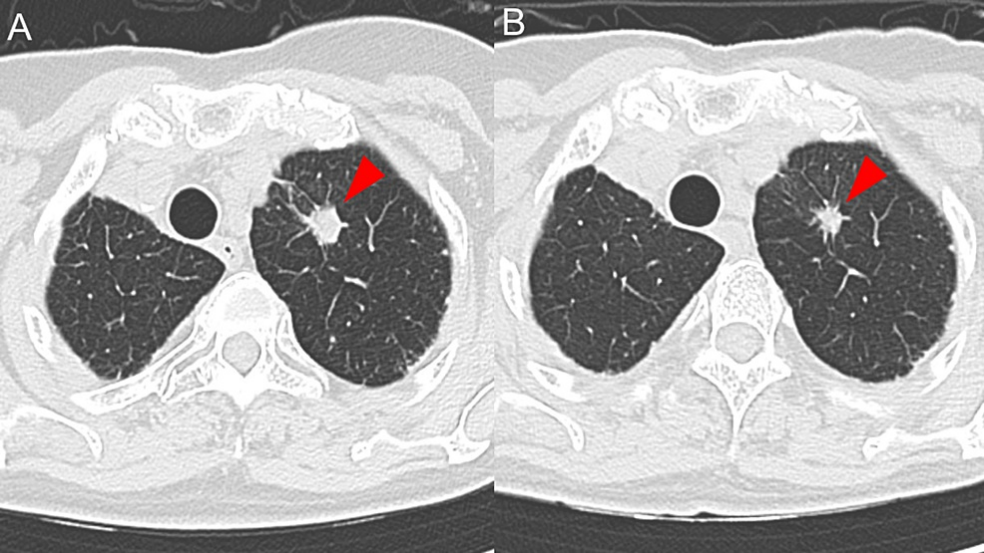
A series of chest computed tomography during trametinib treatment; at the initiation of the treatment (A) and after eight months of trametinib treatment (B), showing partial response.

As a result, trametinib monotherapy was commenced as a third-line treatment. A chest CT scan conducted eight months later showed controlled tumor growth (Figure 4A, 4B).

## Discussion

BRAF fusion gene aberrations in NSCLC are rare, and established treatments for these specific tumors remain undefined. This report presented the first case of an NSCLC patient with SLC44A1-BRAF fusion responding to immunotherapy followed by trametinib.

The RAF protein family plays a crucial role in cellular proliferation signaling, with the BRAF protein being a significant subtype. Predominantly, RAF mutations are linked to the BRAF gene [3], with the V600E mutation comprising approximately half of the BRAF mutations in NSCLC [11]. BRAF possesses three conserved regions (CR): CR1 contains the RAS-binding domain, CR2 is followed by hinge regions containing a cluster of phosphorylation sites that negatively control BRAF signaling, and CR3 encompasses kinase activity through involvement in RAF dimerization [8]. BRAF mutations such as V600E mimic phosphorylation of the activation loop generating a mutation-specific active conformation of the kinase domain which constitutively renders the Ras-Raf-MEK-ERK pathway leading to uncontrolled signaling and tumor growth [8]. The BRAF V600E mutation is a recognized therapeutic target, commonly treated with the BRAF inhibitor dabrafenib in combination with the MEK inhibitor trametinib, yielding substantial efficacy [12]. Furthermore, BRAF mutant NSCLC is associated with elevated PD-L1 expression [13] possibly explained by PD-L1 upregulation by BRAF mutation such as V600E [14]. In fact, immune checkpoint inhibitors have shown favorable efficacy in BRAF mutant NSCLC, indicating that BRAF/MEK inhibitors and immunotherapy could be viable treatment options for these patients [13].

A seminal study by Ross JS et al. reported a pioneering study of 55 cases of tumors carrying BRAF gene fusions from CGP of 20573 tumors across 12 tumor types [6]. All BRAF gene fusions identified in the study were in-frame with breakpoints on the BRAF hotspot range intron 7 to intron 10 and preserved intact BRAF kinase domain. This suggests that the oncogenic mechanisms of BRAF gene fusions differ from those in V600E mutation cases, although their pathomorphological characteristics are similar [15]. Studies on the TTYH3-BRAF fusion protein, an oncogenic driver with unmutated kinase domains, demonstrated its independence from a functional RAS binding domain while showing increased MEK/ERK signaling, which could be inhibited by trametinib [8]. The SLC44A1-BRAF fusion detected in our case has not been reported in the NSCLC public database (COSMIC and TCGA). In the largest screening of BRAF fusion genes, all NSCLC with BRAF fusions were adenocarcinoma or NSCLC with adenocarcinoma features not in squamous or small cell lung cancers as ours [6]. Lind KT et al. reported detecting this rare fusion in a 7-year-old male with low-grade glioma and molecular pathology of this brain tumor noted this fusion juxtaposed the serine-threonine kinase domain of BRAF and SLC44A1 and replaced the 5’ regulatory domain of BRAF while preserving the kinase domain of BRAF [10]. These molecular insights suggest that MEK inhibitor trametinib monotherapy could be a promising approach for NSCLC cases harboring BRAF fusions with intact kinase domains. Although there are limited reports on anti-BRAF targeted therapy for NSCLC with such fusions [9,16,17], trametinib has demonstrated antitumor efficacy in similar cases, including ours. Furthermore, our patient's positive PD-L1 status and response to immunotherapy align with findings in a previous case report [9], even though the association between BRAF gene fusion and response to immunotherapy has not been clearly elucidated unlike in NSCLC harboring BRAF mutations [13].

## Conclusions

In summary, this report described a novel SLC44A1-BRAF fusion in non-small cell lung cancer (NSCLC) showing a favorable response to immunotherapy, followed by trametinib, a downstream MEK inhibitor. Further research is warranted to elucidate the biological and oncogenic mechanisms of NSCLC patients harboring BRAF fusions to refine and expand therapeutic strategies.
